# Differential innervation of superficial versus deep laminae of the dorsal horn by bulbo-spinal serotonergic pathways in the rat

**DOI:** 10.1016/j.ibror.2017.04.001

**Published:** 2017-04-09

**Authors:** A. Gautier, D. Geny, S. Bourgoin, J.F. Bernard, M. Hamon

**Affiliations:** INSERM UMR 894 - Centre de Psychiatrie et Neurosciences, Université Paris Descartes, 75014, Paris, France

**Keywords:** Serotonin, Nucleus raphe magnus, Nucleus reticularis paragigantocellularis pars lateralis, Anterograde labeling, Dorsal horn laminae, Serotonin transporter

## Abstract

Convergent data showed that bulbo-spinal serotonergic projections exert complex modulatory influences on nociceptive signaling within the dorsal horn. These neurons are located in the B3 area which comprises the median raphe magnus (RMg) and the lateral paragigantocellular reticular (LPGi) nuclei. Because LPGi 5-HT neurons differ from RMg 5-HT neurons regarding both their respective electrophysiological properties and responses to noxious stimuli, we used anatomical approaches for further characterization of the respective spinal projections of LPGi *versus* RMg 5-HT neuron subgroups.

Adult Sprague-Dawley rats were stereotaxically injected into the RMg or the LPGi with the anterograde tracer Phaseolus vulgaris leucoagglutinin (PHA-L). The precise location of injection sites and RMg vs LPGi spinal projections into the different dorsal horn laminae were visualized by PHA-L immunolabeling. Double immunofluorescent labeling of PHA-L and the serotonin transporter (5-HTT) allowed detection of serotonergic fibers among bulbo-spinal projections.

Anterograde tracing showed that RMg neurons project preferentially into the deep laminae V-VI whereas LPGi neuron projections are confined to the superficial laminae I-II of the ipsilateral dorsal horn. All along the spinal cord, double-labeled PHA-L/5-HTT immunoreactive fibers, which represent only 5–15% of all PHA-L-immunoreactive projections, exhibit the same differential locations depending on their origin in the RMg *versus* the LPGi.

The clear-cut distinction between dorsal horn laminae receiving bulbo-spinal serotonergic projections from the RMg *versus* the LPGi provides further anatomical support to the idea that the descending serotonergic pathways issued from these two bulbar nuclei might exert different modulatory influences on the spinal relay of pain signaling neuronal pathways.

## Highlights

• Anterograde tracing and 5-HT transporter immunochemistry allowed investigations of bulbo-spinal 5-HT projections in rats.
• 5-HT fibers represent ∼5% vs ∼15% of descending projections from n. raphe magnus vs n. paragigantocellularis, respectively.
• 5-HT fibers from the n. raphe magnus project mainly into dorsal horn laminae V-VI, and to lower extent into laminae I-II.
• In contrast, 5-HT fibers from the n. paragigantocellularis project densely and exclusively into superficial laminae I-II.
• These anatomical data suggest that spinal 5-HT from raphe magnus vs paragigantocellularis n. exert distinct pain controls.

## 1 Introduction

The rostral ventromedial medulla (RVM), which comprises the serotonergic region B3 with serotonin (5-hydroxytryptamine, 5-HT) - containing perikarya in the raphe magnus nucleus (RMg) and the lateral paragigantocellular reticular nucleus (LPGi), is known to play an important role in the bulbo-spinal descending control of nociceptive messages (Fields and Basbaum, 1978, Besson and Chaouch, 1987, Millan, 2002). Accordingly, both RMg and LPGi serotonergic neurons project directly into the dorsal horn of the spinal cord where is located the first central synaptic relay of nociceptive signals generated at peripheral nociceptors (Skagerberg and Bjorklund, 1985, Jones and Light, 1990, Jones and Light, 1992). Furthermore, direct electrical stimulation of the RMg, that produces marked antinociceptive effects (Liebeskind et al., 1973, Oliveras et al., 1974), triggers a concomitant release of 5-HT at the spinal level (Bourgoin et al., 1980, Rivot et al., 1982, Hentall et al., 2006). The causal relationship between spinal 5-HT release and antinociception was clearly demonstrated in studies showing that the antinociceptive effect of RMg electrical stimulation could be suppressed in rats pretreated with the tryptophan hydroxylase inhibitor p-chlorophenylalanine, which produces a marked 5-HT depletion throughout the CNS (Rivot et al., 1980). On the other hand, the selective degeneration of bulbo-spinal serotonergic projections by the neurotoxin 5,7-dihydroxytryptamine was found to significantly alter the nocifensive behaviors associated with inflammatory (Yang et al., 2014) as well as neuropathic pain (Rahman et al., 2006), further confirming the implication of this descending pathway in pain modulatory mechanisms. More recently, novel shRNA interference approaches to selectively deplete 5-HT in these pathways provided clear-cut demonstration of 5-HT-mediated bulbo-spinal modulations of both acute and chronic pain in rats (Wei et al., 2010, Gautier et al., 2017), in convergence with a recent report on the effectiveness of selective optogenetic activation of RVM-serotonergic neurons to markedly affect pain signaling in rats (Cai et al., 2014).

In spite of such a large body of studies devoted to unveil the actual role of bulbo-spinal serotonergic pathways in the control of pain signaling, the situation is still confused to date. Thus, some authors (Suzuki et al., 2004, Rahman et al., 2006, Wei et al., 2010, Guo et al., 2014) concluded that these pathways are implicated in pain promoting mechanisms whereas others (Yamazaki et al., 1999, Hung et al., 2003, Gencer et al., 2015, Lee et al., 2015) reached the opposite conclusion, i.e. these pathways exerting an inhibitory influence on pain signaling.

One possible explanation of such discrepancies might be related to the existence of at least two different serotonergic pathways issued from the B3 region in rats. Indeed, as recalled above, this region comprises two separate clusters of serotonergic neurons, in the RMg and the LPGi, respectively. Interestingly, anatomical and electrophysiological studies provided evidence that 5-HT neurons in the RMg differ to some extent from those in the LPGi. Whereas serotonergic neurons in the RMg receive afferences from the ventral periaqueductal gray (PAG) (Fardin et al., 1984, Cameron et al., 1995, Hermann et al., 1997), those in the LPGi are innervated by projections coming mainly from the dorsal PAG (Fardin et al., 1984, Cameron et al., 1995, Normandin and Murphy, 2008). On the other hand, electrophysiological recordings by Gau et al. (2013) showed that LPGi serotonergic neurons are more intensely activated than RMg serotonergic neurons by thermal noxious stimulation (48–50°C). Accordingly, c-Fos immunolabeling showed a larger number of activated serotonergic neurons in the LPGi than in the RMg in response to thermal noxious stimuli (Gau et al., 2009).

In view of these differences at the level of cell bodies within the B3 region, our aim was to investigate whether differences also exist regarding the respective projections of RMg and LPGi serotonergic neurons into the dorsal horn of the spinal cord in rats. To this aim, we injected an anterograde tracer, the Phaseolus vulgaris leucoagglutinin (PHA-L), specifically into the RMg or the LPGi, and we used anti-PHA-L antibodies to visualize labeled fibers and terminals within dorsal horn laminae. Among these fibers, those with a serotonergic phenotype were characterized by double immunofluorescence labeling with anti-PHA-L and anti-serotonin transporter (5-HTT) antibodies. These investigations allowed the demonstration that specific dorsal horn laminae are reached by bulbo-spinal serotonergic fibers depending on their origin in the RMg or the LPGi, respectively.

## 2 Experimental procedures

### 2.1 Animals

Male Sprague-Dawley rats (body weight: 200–220 g, on arrival) were purchased from Janvier Breeding Center (53940 Le Genest Saint Isle, France). They were housed in a standard controlled environment (22 ± 1°C, 60% relative humidity, 12:12 h light-dark cycle, lights on at 7:00 a.m.) with food and water available *ad libitum*. Animals were allowed to habituate to these housing facilities without any handling for at least 1 week before being used for experiments. In all cases, experiments strictly followed the institutional guidelines that are in compliance with national and international laws and policies for use of animals in neuroscience research (Council Directive 86/609/EEC of the European Communities, November 24, 1986; Council Directive 87-848 of the French Ministère de l’Agriculture et de la Forêt, Service vétérinaire de la santé et de la protection animale, October 19, 1987). Data reported herein were obtained from studies specifically approved under registered nb 01296.02 by the Animal Research Committee of the French Ministère de l’Education Nationale, de l’Enseignement Supérieur et de la Recherche.

### 2.2 Intra-bulbar injection of Phaseolus vulgaris leucoagglutinin (PHA-L)

Surgical procedures were performed in rats anesthetized with a combination of ketamine and xylazine (75 mg/kg and 12.5 mg/kg i.p., respectively) in saline (0.9% NaCl, total volume: 1.5 ml/kg i.p.) and mounted in a stereotaxic frame. The incisor bar was set at 18 mm below the horizontal plane passing through the interaural line, to incline the head by a 30° angle with respect to the horizontal plane at lambda and bregma level (Paxinos and Watson, 2005). With this head position, injections could be easily made along the rostro-caudal extension of B3 area. The scalp was incised at midline and a small opening was made into the atlanto-occipital membrane. PHA-L (Vector Laboratories, Burlingame, CA, USA; 10% in saline) was iontophoretically injected using a glass micropipette mounted with an angle of 45° (with respect to verticality) on the stereotaxic frame. The tip of the micropipette was moved down into the RMg (in 5 rats) or the LPGi (in 6 rats), at a point 200 μm posterior - 800 μm anterior or 400 μm posterior - 400 μm anterior to the obex, and 0 or 1200 μm lateral (on the right side) to the mid-sagittal plane, respectively. PHA-L solution was outflowed by direct current (3–5 μA) passing through the micropipette with a 15 s on/off cycle for 15 min. Once injection was achieved, the scalp was sewed up, and animals returned to their home cages after recovery from anesthesia.

### 2.3 Immunohistochemistry

#### 2.3.1 Tissue preparation

Following a postoperative survival time of 20 days, rats were deeply anesthetized with sodium pentobarbital (50 mg/kg i.p.) and perfused transcardially with a heparin (25 IU/ml)-saline solution (during 3 min), followed by 0.12 M phosphate buffered saline (PBS, pH 7.4) containing 4% paraformaldehyde, 0.1% glutaraldehyde and 0.05% picric acid for 25 min, and finally by 20% sucrose solution (during 10 min). The brain and spinal cord were removed and cryoprotected in 20% sucrose solution overnight. The next day, coronal sections of 50 μm thickness were cut using a freezing microtome and collected in containers filled with PBS. Four series of brain stem and spinal cord sections (at cervical C5, thoracic T5, lumbar L4-5, and sacral S3 levels) were made for each rat. They were processed in parallel with the same reagents, under the very same conditions for immunohistochemical labelings.

#### 2.3.2 Immunohistochemical procedures

For PHA-L immunolabeling, sections were preincubated for 1 h in PBS containing 0.4% Triton X-100 and 1% normal goat serum (NGS), then incubated overnight with the primary antibody (rabbit anti-PHA-L, 1/2,000, AB_2313686, Vector Laboratories, Burlingame, CA, USA). After rinsing for 30 min with PBS, sections were incubated for 1 h with the secondary antibody (goat biotinylated anti-rabbit, 1/200, AB_2313606, Vector) in PBS containing 0.4% Triton X-100 and 1% NGS, then rinsed with PBS and incubated for 1 h in the avidin-biotin-horseradish-peroxidase solution (ABC Vectastain Elite Kit, Vector Laboratories). Sections were then processed in two series. The first series was stained (brown/orange) with 0.4% 3,3’-diaminobenzidine (DAB, Sigma-Aldrich, St Louis, MO, USA) in PBS supplemented with H_2_O_2_ at progressively increasing concentrations (0.00025%, 0.0005%, 0.001%, 0.002%, 0.004%). The second series was first rinsed with 0.1 M Tris-HCl (pH 7.4, 15 min) and then stained (black/gray) with 0.4% DAB (Sigma-Aldrich) + 2% ammonium nickel sulfate in Tris buffer supplemented with increasing concentrations of H_2_O_2_ as described above. After rinsing in respective buffer, sections on glass slides were cover-slipped with Eukitt (Sigma-Aldrich).

#### 2.3.3 Immunofluorescence procedure

For the double immunofluorescence labeling of PHA-L and 5-HTT, spinal cord sections were preincubated for 1 h in PBS containing 0.4% Triton X-100, 2% bovine serum albumin (BSA) and 1% normal donkey serum. Sections were then incubated overnight at 4°C with rabbit anti-PHA-L (1/500, Vector) and goat anti-5-HTT (1/2000, AB_632391, Santa Cruz Biotechnology Inc, Paso Robles, CA, USA) antibodies in the same buffer. After rinsing for 30 min with PBS, sections were incubated for 90 min with horse biotinylated anti-goat antibodies (1/200, AB_2336123, Vector) in PBS containing 0.4% Triton X-100, 2% BSA and 1% normal donkey serum. Then, after rinsing for 30 min with PBS, the sections were incubated for a further 90 min with donkey anti-rabbit Alexa 594 fluorescent antibodies (1/1,000, AB_141637, Life Technologies, Carlsbad, CA, USA) and streptavidin Alexa 488 (1/2,000, AB_2336881, Life Technologies). After final extensive rinsing in PBS buffer, sections on glass slides were coverslipped with Vectashield (Vector).

### 2.4 Data acquisition, analysis and reconstruction

The RMg and LPGi nuclei in B3 area, and the spinal subdivisions were defined according to Paxinos and Watson (2005). The laminar distribution of spinal PHA-L projections of RMg *versus* LPGi neurons was examined by considering three distinct gray matter regions in the dorsal horn: the superficial laminae (laminae I-II), the nucleus proprius (laminae III-IV) and the neck (laminae V-VI).

#### 2.4.1 Photomicrographs under brightfield illumination

Immunolabeling was analyzed in the two different series of coronal sections (200 μm apart) processed with DAB only or DAB-nickel for the PHA-L injection sites in the RMg and the LPGi or PHA-L ir fibers in the spinal cord, respectively. Digitized photomicrographs were made using a computer-assisted procedure as described in details elsewhere (Gau et al., 2013). Briefly, a CCD color video camera, connected to an Olympus BX51 microscope, sent an RGB (Red, Green, Blue) output to a Macintosh computer. Images at different focal planes were captured and digitized with a 24 bit color-scale using Openlab software (Improvision, Coventry, UK). An operator allowed the combination, pixel-by-pixel, of images in different focal planes. These operations resulted in the production of one image by incorporating the darkest value of each corresponding pixel in each focal plane for each of red, green and blue color plan. Images were exported to Adobe Photoshop (version CS5) software in order to mount adjacent digitized images in the same plane for making a final high resolution and large field image. After adjustment of brightness, contrast and image scale, additional indications and anatomical landmarks were incorporated to the final figure.

#### 2.4.2 Reconstruction of the brainstem

To document the locations of RMg or LPGi injection sites, drawings based upon the morphology of brainstem sections were used. These drawings were digitized using Adobe Illustrator (version CS5) software as follows: digitized standard atlas brainstem sections from Paxinos and Watson (2005) were firstly used, then superimposed on coronal sections at corresponding level from several rats and finally line drawings were adjusted to the outline of the different sections. Anatomical landmarks and nomenclature according to Paxinos and Watson (2005) were finally incorporated to the resulting figure.

#### 2.4.3 Fluorescent illumination

##### 2.4.3.1 Confocal images

Immunofluorescence images, from PHA-L/5-HTT double labeling of spinal cord sections, were generated using a Leica TCS SP5 AOBS laser scanning confocal microscope (40× oil-immersion lens). The images were captured digitally using Leica LAS AF software. All acquisitions were made using the very same setup for the collection of a stack of images of 10 μm thickness, taken every 1.01 μm (Z resolution) with a XY resolution of 0.379 μm per pixel. Leica pictures were saved as LIFF images and then used for the quantitative analysis or as assembled TIFF images for illustration using Adobe Photoshop (version CS5) software. Contrast and brightness of images displayed in figures were adjusted using Adobe Photoshop (version CS5) software, only for illustrations.

##### 2.4.3.2 Quantitative analysis of serotonergic fibers in spinal cord sections

In order to process the confocal z-stack images for quantitative analysis, a mask was firstly generated on Image J software by using a combination of maximum filter and intermodes threshold for the green channel and median filter and intermodes threshold for the red channel corresponding to 5-HTT and PHA-L labeling, respectively. The mask was then superimposed to the raw images, without impact on the fibers'integrity, in order to reduce background noise and allow distinct visualization and quantification of the signal of interest. The volume (μm^3^) and diameter (μm) of labeled fibers were measured, within specific region of interest for each spinal cord section corresponding to laminae I-II and V-VI, on 3D reconstruction confocal images of 5-HTT, PHA-L and merge immunofluorescence using Volocity software (Version 6.3, PerkinElmer). The percentage of serotonergic fibers among PHA-L labeled fibers issued from the RMg *versus* the LPGi nucleus was estimated from the ratio of the volume of double-labeled over that of total PHA-L-labeled fibers in the laminae V-VI *versus* I-II in the respective groups of PHA-L injected rats.

### 2.5 Statistical analyses

All values for the quantitative characterization of the different categories of immunoreactive (ir) fibers are expressed as means ± S.E.M. of independent determinations in 5–6 rats per group. The unpaired Mann-Whitney test was used to assess significant differences at *P* ≤ 0.05.

## 3 Results

### 3.1 PHA-L injection sites in the nucleus raphe magnus and the lateral paragigantocellular reticular nucleus

**Fig. 1:**
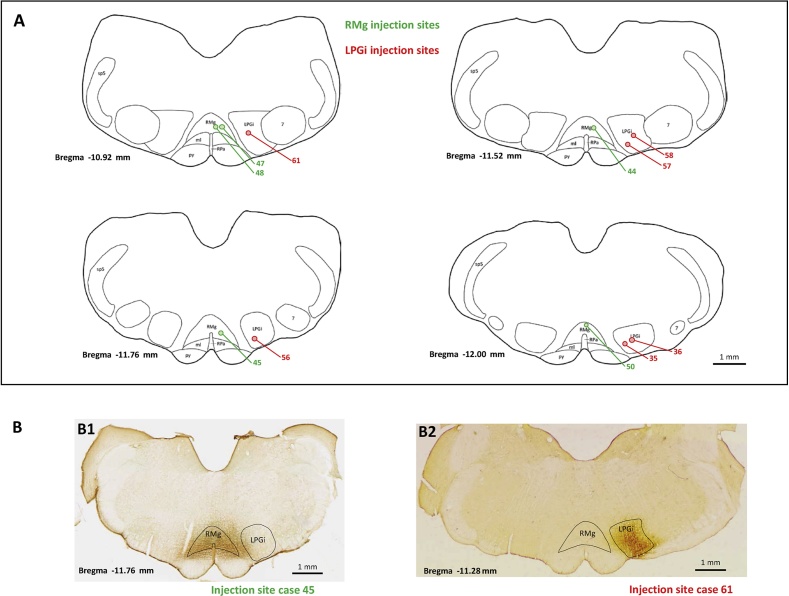
Typical photomicrographs and distributions of PHA-L injection sites within the RMg or the LPGi. PHA-L immunolabeling was performed 20 days after PHA-L injection into either of these two nuclei in the right B3 area. A - Schematic drawings of coronal sections at −10.92/11.52/11.76/12.00 mm from bregma (Paxinos and Watson, 2005) with the locations of RMg injection sites (in green) in 5 rats and LPGi injection sites (in red) in 6 rats. (B) Photomicrographs of typical PHA-L immunolabeling in the RMg (B1, case 45: interaural −2.76 mm, bregma −11.76 mm) or the LPGi (B2, case 61: interaural −2.28 mm, bregma −11.28 mm; coordinates according to Paxinos and Watson, 2005) showing the specific immunolabeling of each nucleus. Scale bar = 1 mm. Abbreviations = 7: nucleus facialis; LPGi: nucleus reticularis paragigantocellularis, pars lateralis; ml: medial lemniscus; py: pyramidal tract; RMg: nucleus raphe magnus; RPa: nucleus raphe pallidus; sp5: spinal nucleus of the trigeminal nerve.

Typical PHA-L immunolabeling after injection into the RMg or the LPGi is illustrated in Fig. 1B1 and 1B2, respectively. The distribution of all injection sites within the RMg (n = 5) and the LPGi (n = 6) is represented in the sections drawings shown in Fig. 1A. For the sake of clarity, only 2–3 injection sites (one injection per rat) are represented on each section with reference to the bregma. In all of the 11 injected rats selected for thorough analyses, injection was made precisely into the targeted site, and PHA-L diffusion was very limited, allowing neuron labeling mainly confined to either the RMg or the LPGi (on the injected right side; Fig. 1B1, B2). In case of the RMg, which is located in the sagittal plane, unilateral injection of PHA-L (on the right side) could not avoid diffusion into the contralateral part of the nucleus (see case 45, Fig. 1B). However, only minor PHA-L spread, if any, to the LPGi could be visualized in all RMg-injected rats (Fig. 1A,B1). Visualization of serial sections showed that PHA-L immunolabeling extended caudo-rostrally over 1000–1500 μm, which covered most of the caudo-rostral extension of RMg or LPGi nucleus (on the injected right side; not shown).

### 3.2 Respective distributions of PHA-L-ir fibers in the spinal cord in rats injected with PHA-L into the RMg versus the LPGi

**Fig. 2:**
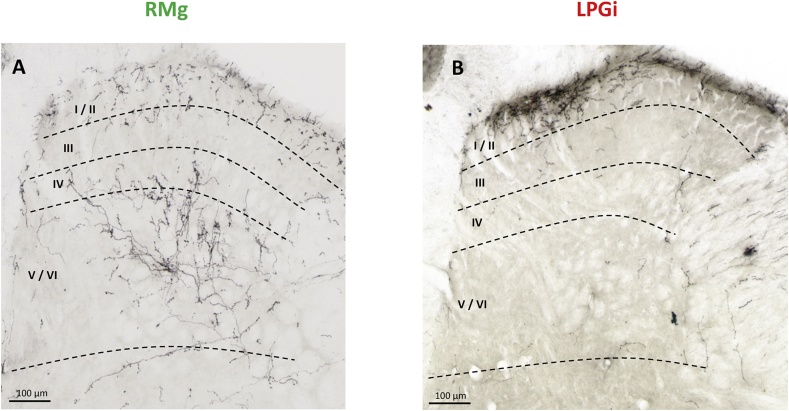
Typical photomicrographs of PHA-L-immunolabeled fibers within the right lumbar (L5) dorsal horn in rats injected with PHA-L into the RMg (A) or ipsilateral LPGi (B). PHA-L immunolabeling was performed 20 days after intra-RMG or intra-LPGi injection. (A) PHA-L-immunoreactive fibers issued from the RMg are located mainly in deep laminae V-VI and to a lower extent in superficial laminae I-II. (B) PHA-L immunoreactive fibers from the LPGi are especially dense in laminae I-II and not detected in other laminae. Similar pictures were obtained in 5 (A-RMg) and 6 (B-LPGi) injected rats. Scale bar = 100 μm.

Representative digitized photomicrographs of PHA-L immunolabeling in spinal cord sections at lumbar level are shown in Fig. 2. As no immunolabeling was found in the ventral half of the spinal cord (not shown), photomicrographs are focused on the dorsal half only. In rats which had been injected with PHA-L precisely into the RMg, PHA-L-ir fibers were mainly found in the deep laminae V-VI of the dorsal horn, and an additional relatively faint immunolabeling associated with shorter fiber segments was detected within the superficial laminae I-II as shown in the typical example illustrated in Fig. 2A. In contrast, the other typical example illustrated in Fig. 2B shows that a dense network of PHA-L-ir fibers was detected only in the latter laminae on the ipsilateral (right) side in rats which had been injected with PHA-L precisely into the right LPGi. In all of the 6 rats injected into the LPGi, no PHA-L-ir fibers were found within the deep laminae of the dorsal horn (Fig. 2B). Thus, the very same differential distributions of PHA-L-ir fibers were found in the 11 PHA-L-injected rats. PHA-L-ir fibers were distributed mainly within laminae V-VI and at a lower density in laminae I-II in the 5 rats injected into the RMg, and at a high density exclusively within the latter superficial laminae in the 6 rats injected into the LPGi.

**Fig. 3:**
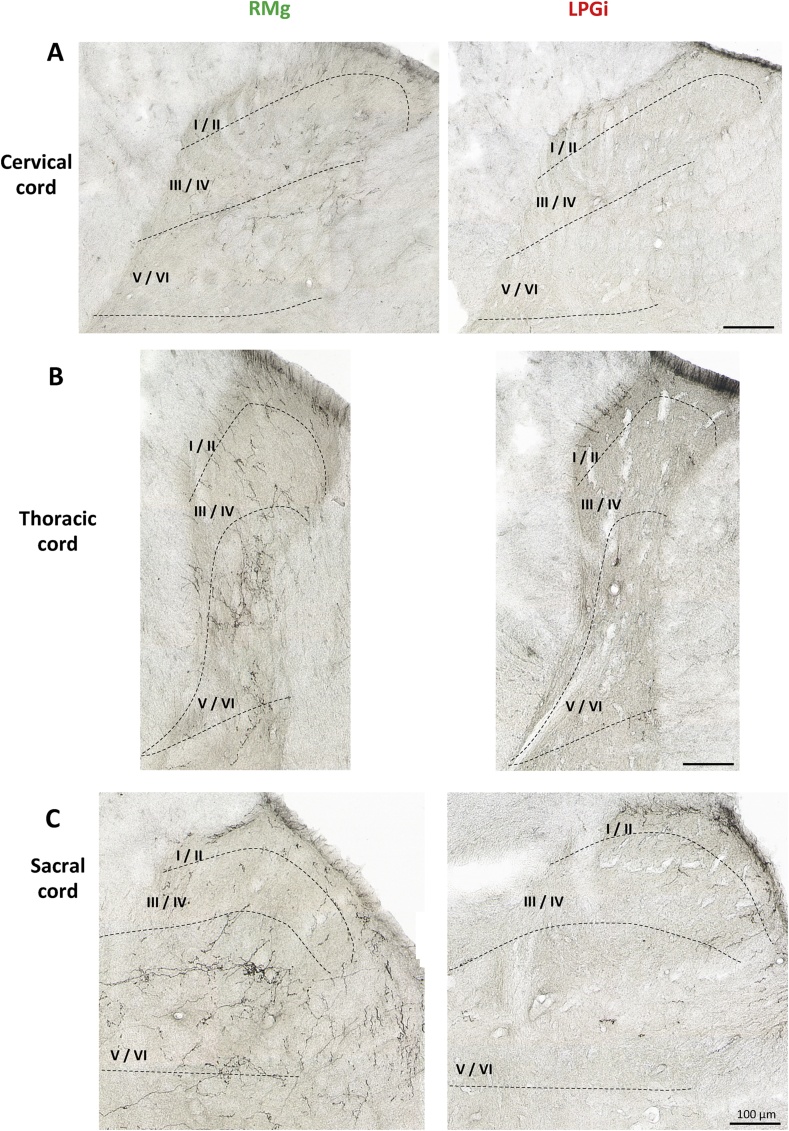
Typical photomicrographs of PHA-L-immunolabeled fibers at cervical (A), thoracic (B) and sacral (C) levels of the spinal cord on the right side in rats injected with PHA-L into the RMg or ipsilateral LPGi. Like that observed at lumbar level (Fig. 2), spinal cord sections at cervical (C5), thoracic (T5) as well as sacral (S3) level show that PHA-L-immunolabeled projections from the RMg are distributed within the superficial laminae I-II and the deep laminae V-VI of the dorsal horn. In contrast, those from the LPGi are confined, at a high density, within the superficial laminae I-II exclusively. Scale bar = 100 μm.

Examination of PHA-L-ir in sections from other spinal cord segments showed that the differential distribution observed at the lumbar level was also found all along the spinal cord extension. In particular, in both thoracic T5 (Fig. 3B) and sacral S3 (Fig. 3C) segments, PHA-L-ir fibers in rats injected with the anterograde tracer into the LPGi were observed exclusively within the superficial laminae I-II. In rats injected into the RMg, PHA-L-ir fibers were also found in the latter laminae but a dense network was particularly visualized in the deep laminae V-VI (Fig. 3B and C). At the cervical C5 level (Fig. 3A), PHA-L immunolabeling was less intense than in thoracic, lumbar (Fig. 2) and sacral segments but PHAL-ir fibers also exhibited the same distributions, i.e. within both the superficial I-II and deep V-VI laminae in RMg injected rats *versus* only the superficial I-II laminae in LPGi injected rats.

### 3.3 Characteristics of serotonergic fibers among PHA-L-ir projections from PHA-L-injected neurons in RMg versus LPGi

**Fig. 4:**
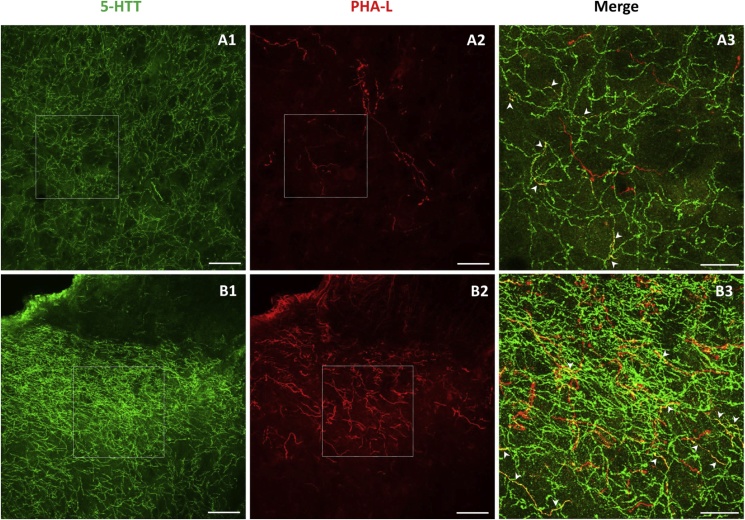
Typical photomicrographs of 5-HTT- and PHA-L-immunofluorescent fibers within the right dorsal horn of the lumbar cord 20 days after PHA-L injection into the RMg (A) or ipsilateral LPGi (B). A1, B1: Immunofluorescent labeling of 5-HTT-ir fibers, in green, in laminae V and IIo, respectively. A2, B2: Immunofluorescent labeling of PHA-L-ir fibers, in red, in the same sections as in A1 and B1. A3, B3: Superimposition of 5-HTT- and PHA-L immunolabeling in the squares outlined by white dotted lines in the section A1,B1: A3, and in the section B1,B2: B3. Co-localization of both immunolabelings (in yellow) is present in fibers pointed by white arrow heads. Scale bar = 50 μm in A1,A2,B1,B2; 20 μm in A3,B3.

Immunofluorescent labeling of 5-HTT in lumbar cord sections showed a dense network of immunolabeled fibers within both the superficial laminae I-II (Fig. 4B1) and deep laminae V-VI (Fig. 4A1) of the dorsal horn, as expected from the known distribution of spinal 5-HT innervation in rats (Steinbusch, 1981, Marlier et al., 1990). Double immunofluorescent labeling of PHA-L in red (Fig. 4, A2, B2) and 5-HTT in green (Fig. 4, A1, B1) in lumbar cord sections allowed the visualization of fibers endowed with superimposed immunolabelings (in yellow) in both the deep V-VI laminae (Fig. 4, A3) and superficial I-II (mainly IIo) laminae (Fig. 4, B3). Double 5-HTT/PHA-L-ir fibers from PHA-L-injected RMg neurons were found to extend on a relatively long distance with vertical, horizontal or oblique orientations throughout the deep V-VI laminae (Fig. 4, A3). In contrast, double 5-HTT/PHA-L-labeled fibers from PHA-L-injected LPGi neurons were short, transversely oriented and confined within the superficial laminae I-II (Fig. 4, B3). The same differential distributions of double labeled 5-HTT/PHA-L fibers as those illustrated in Fig. 4 for the lumbar dorsal horn were also observed at the cervical, thoracic and sacral levels (not shown).

Further characterization of serotonergic fibers within the bulbo-spinal PHA-L-ir projections indicated that their average diameter, of ∼1.20 μm, was slightly less (−8 to −11%) than the average value for the whole PHA-L-ir fibers (Table 1). Quantification of the double labeled (yellow) fiber volume over the PHA-L labeled (red) one within laminae I-II in LPGi-injected rats compared to laminae V-VI in RMg-injected rats (see Fig. 4 A3,B3) showed that serotonergic fibers represented a limited population (5–20%) among PHA-L-ir bulbo-spinal projections, especially within deep laminae where their relative density reached only 30% of that in superficial laminae (Table 1). These characteristics were essentially similar at the cervical, thoracic, lumbar and sacral levels of the rat spinal cord (Table 1).

**Table 1:** Characteristics of PHA-L - and 5-HTT-immunoreactive (ir) fibers within the dorsal horn in rats injected with PHA-L into the LPGi or the RMG. The diameter (in μm) of respective fibers and the density ([5-HTT-ir + PHA-L-ir]/[ PHA-L-ir] as percentage) of 5-HTT-ir fibers among the whole population of PHA-L-ir fibers were determined at the cervical, thoracic, lumbar and sacral levels in the right dorsal horn of rats injected with PHA-L into the ipsilateral LPGi or RMg 20 days before sacrifice (see Experimental Procedures).

Fibers' characteristics	Spinal level	Laminae I-II (from LPGi)	Laminae V-VI (from RMg)
Diameter of PHA-L-ir fibers (μm)	Cervical (C5)	1.26 ± 0.01	1.25 ± 0.02
	Thoracic (T5)	1.24 ± 0.02	1.27 ± 0.03
	Lumbar (L5-6)	1.28 ± 0.01	1.32 ± 0.04
	Sacral (S3)	1.26 ± 0.03	1.33 ± 0.02
Diameter of 5-HTT-ir fibers (μm)	Cervical (C5)	1.19 ± 0.02	1.13 ± 0.01
	Thoracic (T5)	1.18 ± 0.03	1.15 ± 0.03
	Lumbar (L5-6)	1.17 ± 0.02	1.17 ± 0.01
	Sacral (S3)	1.20 ± 0.03	1.15 ± 0.01
[5-HTT-ir + PHA-L-ir]/[ PHA-L-ir] %	Cervical (C5)	16.47 ± 3.04	4.65 ± 1.36*
	Thoracic (T5)	19.62 ± 4.62	4.88 ± 2.88*
	Lumbar (L5-6)	15.55 ± 3.68	4.80 ± 1.01*
	Sacral (S3)	16.74 ± 4.94	4.81 ± 1.34*

Each value is the mean ± S.E.M. of independent determinations in 5 (RMg) or 6 (LPGi) rats.

*P < 0.05 versus laminae I-II, Mann-Whitney test.

## 4 Discussion

In the present study, we used an anterograde tracer, PHA-L, to label the bulbo-spinal pathways and investigate the anatomical organization of the descending projections from the serotonergic B3 region within the RVM. More specifically, we focused on the respective projections from the RMg and the LPGi nuclei, two structures in the B3 region which were previously shown to contain serotonergic neurons innervating the spinal cord (Fields and Basbaum, 1978, Besson and Chaouch, 1987). This approach provided a more specific characterization of these bulbo-spinal pathways from the RMg *versus* the LPGi than that achieved from previous anatomical studies. Indeed, previous anterograde tracings were made from tracer injection into the raphe magnus (and pallidus) nuclei (Jones and Light, 1990), which did not allow any identification of spinal projections specifically from the LPGi. Similarly, studies aimed at retrograde labeling (Skagerberg and Bjorklund, 1985, Jones and Light, 1992, VanderHorst and Ulfhake, 2006) consisted of injecting the tracer into the whole dorsal horn, which did not allow any distinction of the different projections innervating specifically the different laminae.

By injecting PHA-L specifically into each of the two serotonergic nuclei of the B3 region, we showed here that the RMg neurons project preferentially into the deep laminae V-VI of the dorsal horn of the spinal cord whereas the LPGi neurons send projections exclusively into the superficial laminae I-II. Identification of serotonergic fibers among these projections was made using the 5-HT neuron biomarker, 5-HTT (Blakely et al., 1994), because previous studies showed that anti-5-HTT antibodies, much more than anti-5-HT antibodies, yielded satisfactory immunolabeling of 5-HT fibers (Fournet et al., 2010). Thus, double immunofluorescent labeling of 5-HTT and PHA-L allowed the demonstration that part of PHA-L fibers from both the RMg and the LPGi belong to neurons with a serotonergic phenotype, in agreement with previous reports (Basbaum et al., 1988, Hökfelt et al., 2000, Jones and Light, 1990, Jones and Light, 1992). Quantitative characterization of the latter fibers showed that they are slightly thinner than the whole population of PHA-L-ir fibers, with an average diameter of ∼1.20 μm, typical of unmyelinated fibers (Basbaum et al., 1988). On the other hand, estimation of the relative density of double labeled PHA-L/5-HTT-ir fibers indicated that serotonergic fibers represent only a limited portion of bulbo-spinal projections from the B3 area. Indeed, only ∼5% of PHA-L-ir fibers in rats injected with the anterograde tracer into the RMg were found to express the 5-HTT in laminae V-VI. This percentage reached 15–20% in the superficial laminae I-II where PHA-L-ir fibers originate mainly from the LPGi, suggesting a larger impact of serotonergic projections at this level. In line with this inference, previous retrograde labeling studies concluded that bulbo-spinal serotonergic projections from the LPGi are more numerous than from the RMg (VanderHorst and Ulfhake, 2006). Interestingly, previous quantification of serotonergic neurons within the RVM showed that they represent only 15–18% of the local neuronal population (Potrebic et al., 1994). Such relatively low percentages of both 5-HT cell bodies (Potrebic et al., 1994) and fibers (Table 1) clearly emphasize that the serotonergic projections from the B3 area are only a relatively minor part of the complex (GABAergic, cholinergic, peptidergic …) bulbo-spinal descending systems implicated in the modulation of dorsal horn neuronal activities controlling various physiological functions (triggered by various sensory messages from cutaneous, muscle and visceral afferents) in addition to nociception (Kachidian et al., 1991, Millan, 2002, Chen et al., 2013, Huma et al., 2014).

That spinal serotonergic projections of LPGi neurons appeared confined within the superficial laminae I-II of the dorsal horn whereas those of RMg neurons were visualized mainly in deep laminae V-VI is in line with previous physiological studies (Gau et al., 2013, Gautier et al., 2017) supporting the idea that B3 serotonergic neurons might be differently implicated in pain signaling modulations depending on their location in the RMg *versus* the LPGi. Interestingly, both nuclei received projections from the dorsal raphe nucleus (Vertes and Kocsis, 1994), and it would be of interest to investigate their respective contributions to pain control mechanisms triggered from this more rostral nucleus (Gebhart and Randich, 1990, Wang and Nakai, 1994).

Unmyelinated nociceptive C afferent fibers are known to innervate mostly the superficial dorsal horn (laminae I-IIo) (D'Mello and Dickenson, 2008, Basbaum et al., 2009, Todd, 2010) whereas nociceptive myelinated Aδ afferent fibers project mainly into lamina I and deep lamina V (Basbaum et al., 2009, Cordero-Erausquin et al., 2016). In contrast, the tactile myelinated afferent fibers, i.e. the low-threshold mechanoreceptors Aβ, project into deep laminae III-V of the dorsal horn (Basbaum et al., 2009, Cordero-Erausquin et al., 2016). This lamina specificity suggests that the serotonergic LPGi neurons might modulate the activity of spinal nociceptive neurons mainly in laminae I-II, where are received intense nociceptive messages carried from the periphery via the nociceptive afferent Aδ and C fibers. On the other hand, the serotonergic RMg neurons might impact not only laminae I-II neurons but also – possibly to a larger extent - wide dynamic range (WDR) neurons in lamina V and non-nociceptive neurons in laminae III-V, i.e. neurons receiving few secondary nociceptive information (via mostly the lamina V) (D'Mello and Dickenson, 2008, West et al., 2015, Cordero-Erausquin et al., 2016).

Considering the different supra-spinal projections of neurons in laminae I-II *versus* laminae V-VI, one can also infer that LPGi and RMg serotonergic neurons might be involved in specific, sensory discriminative and/or affective and/or autonomic components of pain. Indeed nociceptive neurons in the lamina I project into the lateral parabrachial area connected with the hypothalamus, the amygdala, the periaqueductal gray and the thalamus. On the other hand, neurons in the lamina V send projections into the reticular nucleus and the medial parabrachial complex, and both laminae I and V neurons relay nociceptive messages to the ventro-postero-lateral nucleus of the hypothalamus via the lateral spinothalamic tract (Bernard and Villanueva, 2009, West et al., 2015).

Such (at least partly) distinct locations of spinal projections of RMg-5-HT neurons *versus* LPGi-5-HT neurons might also underlie some differential capacity of each of these groups of serotonergic neurons to modulate allodynia and hyperalgesia in case of neuropathic pain. Punctate hyperalgesia, resulting in exacerbated pain response to noxious stimuli, intervenes through activity of Aδ fibers (Ziegler et al., 1999) and partly of C fibers (Kilo et al., 1994), which contact nociceptive specific neurons located in laminae I-IIo (Todd, 2010). After peripheral nerve injury, neuroplastic changes in low-threshold afferent mechanosensitive Aβ fibers (Nitzan-Luques et al., 2011, Nitzan-Luques et al., 2013) contribute to tactile allodynia. Indeed modified Aβ fibers can send collaterals from deep laminae III-V into laminae I-II and activate protein kinase C-gamma (PKC-γ)-containing interneurons which excite nociceptive neurons within the latter superficial laminae (West et al., 2015, Cordero-Erausquin et al., 2016), and such anatomical reorganization could contribute to neuropathic tactile pain hypersensitivity and allodynia (Bester et al., 2000, Woolf and Salter, 2000, Todd, 2010, Arcourt and Lechner, 2015, Cordero-Erausquin et al., 2016). Because of the deep laminae location of their projecting fibers, RMg serotonergic neurons, but not LPGi serotonergic neurons, might be involved in some modulation of mechanisms underlying allodynia through neuroplastic changes of Aβ fibers within these laminae. Although our study focused on descending controls of pain signaling mechanisms, it has to be reminded that bulbo-spinal serotonergic pathways also control non-nociceptive signaling within the dorsal horn, notably through 5-HT-mediated modulations of spinal interneurons. In particular, A-fiber inputs generated by weak tactile stimuli or originating from muscle spindle and tendon organ afferents as well as viscera are under the control of bulbo-spinal serotonergic pathways (Carstens et al., 1981, Jankowska et al., 2000), but the respective roles of those coming from the RMg *versus* the LPGi remain to be investigated.

In conclusion, the serotonergic RMg neurons send projections mainly into the deep laminae V-VI of the dorsal horn of the spinal cord whereas the serotonergic LPGi neurons send their projections exclusively into the superficial laminae I-II. Such differential anatomical organization suggests that 5-HT LPGi neurons more than 5-HT RMg neurons might be concerned by modulatory controls of nociceptive signaling triggered by strong noxious stimulation, as previously proposed by Gau et al. (2013). Furthermore, the respective distributions of projections from LPGi *versus* RMg serotonergic neurons also suggest that these neurons might be differentially involved in modulatory controls of allodynia *versus* hyperalgesia under neuropathic pain conditions. Altogether, our data provide a better knowledge of bulbo-spinal serotonergic anatomical organization, and open new perspectives toward investigations of the respective functional implications of 5-HT neurons within the LPGi *versus* the RMg.

## Author contributions

A.G., J.F.B and M.H. designed the study. A.G. and J.F.B. performed the experiments, and A.G. and D.G. quantified the immunolabelings. A.G., S.B., J.F.B. and M.H. analyzed the data, made the illustrations and wrote the manuscript. All authors contributed to editing the manuscript.

## Conflict of interest

All authors declare no conflict of interest.

## Funding

This work was supported by grants from the Institut National de la Santé et de la Recherche Médicale (INSERM), University Pierre and Marie Curie and University Paris Descartes. A.G. was supported by fellowships from the Agence Nationale de la Recherche (ANR, contract 11 BSV4 017 04) during performance of this work. The funders had no role in study design, data collection and analysis, decision to publish, or preparation of the manuscript.

## References

[bib1] Arcourt A., Lechner S.G. (2015). Peripheral and spinal circuits involved in mechanical allodynia. Pain.

[bib2] Basbaum A.I., Bautista D.M., Scherrer G., Julius D. (2009). Cellular and molecular mechanisms of pain. Cell.

[bib3] Basbaum A.I., Zahs K., Lord B., Lakos S. (1988). The fiber caliber of 5-HT immunoreactive axons in the dorsolateral funiculus of the spinal cord of the rat and cat. Somatosens. Mot. Res..

[bib4] Besson J.M., Chaouch A. (1987). Descending serotoninergic systems. Pain Head..

[bib5] Bester H., Beggs S., Woolf C.J. (2000). Changes in tactile stimuli-induced behavior and c-Fos expression in the superficial dorsal horn and in parabrachial nuclei after sciatic nerve crush. J. Comp. Neurol..

[bib6] Bernard J.F., Villanueva L., Bouhassira D., Calvino B. (2009). Architecture fonctionnelle des systèmes nociceptifs. Douleurs : physiologie, physiopathologie et pharmacologie.

[bib7] Blakely R.D., De Felice L.J., Hartzell H.C. (1994). Molecular physiology of norepinephrine and serotonin transporters. J. Exp. Biol..

[bib8] Bourgoin S., Oliveras J.L., Bruxelle J., Hamon M., Besson J.M. (1980). Electrical stimulation of the nucleus raphe magnus in the rat. Effects on 5-HT metabolism in the spinal cord. Brain Res..

[bib9] Cai Y.Q., Wang W., Hou Y.Y., Pan Z.Z. (2014). Optogenetic activation of brainstem serotonergic neurons induces persistent pain sensitization. Mol. Pain.

[bib10] Cameron A.A., Khan I.A., Westlund K.N., Willis W.D. (1995). The efferent projections of the periaqueductal gray in the rat: a Phaseolus vulgaris-leucoagglutinin study. II. Descending projections. J. Comp. Neurol..

[bib11] Carstens E., Bihl H., Irvine D.R.F., Zimmermann M. (1981). Descending inhibition from medial and lateral midbrain of spinal dorsal horn neuronal responses to noxious and nonnoxious cutaneous stimuli in the cat. J. Neurophysiol..

[bib12] Chen T., Wang X.L., Qu J., Wang W., Zhang T., Yanagawa Y., Wu S.X., Li Y.Q. (2013). Neurokinin-1 receptor-expressing neurons that contain serotonin and gamma-aminobutyric acid in the rat rostroventromedial medulla are involved in pain processing. J. Pain.

[bib13] Cordero-Erausquin M., Inquimbert P., Schlichter R., Hugel S. (2016). Neuronal networks and nociceptive processing in the dorsal horn of the spinal cord. Neuroscience.

[bib14] D'Mello R., Dickenson A.H. (2008). Spinal cord mechanisms of pain. Br. J. Anesth..

[bib15] Fardin V., Oliveras J.L., Besson J.M. (1984). Projections from the periaqueductal gray matter to the B3 cellular area (nucleus raphe magnus and nucleus reticularis paragigantocellularis) as revealed by the retrograde transport of horseradish peroxidase in the rat. J. Comp. Neurol..

[bib16] Fields H.L., Basbaum A.I. (1978). Brainstem control of spinal pain-transmission neurons. Ann. Rev. Physiol..

[bib17] Fournet V., Jany M., Fabre V., Chali F., Orsal D., Schweitzer A., Andrieux A., Messanvi F., Giros B., Hamon M., Lanfumey L., Deloulme J.C., Martres M.P. (2010). The deletion of the microtubule-associated STOP protein affects the serotonergic mouse brain network. J. Neurochem..

[bib18] Gau R., Sevoz-Couche C., Hamon M., Bernard J.F. (2013). Noxious stimulation excites serotonergic neurons: a comparison between the lateral paragigantocellular reticular and the raphe magnus nuclei. Pain.

[bib19] Gau R., Sevoz-Couche C., Laguzzi R., Hamon M., Bernard J.F. (2009). Inhibition of cardiac baroreflex by noxious thermal stimuli: a key role for lateral paragigantocellular serotonergic cells. Pain.

[bib20] Gautier A., El Ouaraki H., Bazin N., Salam S., Vodjdani G., Bourgoin S., Pezet S., Bernard J.F., Hamon M. (2017). Lentiviral vector-driven inhibition of 5-HT synthesis in B3 bulbo-spinal serotonergic projections – Consequences on nociception, inflammatory and neuropathic pain in rats. Exp. Neurol..

[bib21] Gebhart G.F., Randich A., Klemm W.R., Vertes R.P. (1990). Brainstem modulation of nociception. Brainstem Mechanisms of Behavior.

[bib22] Gencer A., Gunduz O., Ulugol A. (2015). Involvement of descending serotonergic and noradrenergic systems and their spinal receptor subtypes in the antinociceptive effect of dipyrone. Drug Res. (Stuttg.).

[bib23] Guo W., Miyoshi K., Dubner R., Gu M., Li M., Liu J., Yang J., Zou S., Ren K., Noguchi K., Wei F. (2014). Spinal 5-HT_3_ receptors mediate descending facilitation and contribute to behavioral hypersensitivity via a reciprocal neuron-glial signaling cascade. Mol. Pain.

[bib24] Hentall I.D., Pinzon A., Noga B.R. (2006). Spatial and temporal patterns of serotonin release in the rat's lumbar spinal cord following electrical stimulation of the nucleus raphe magnus. Neuroscience.

[bib25] Hermann D.M., Luppi P.H., Peyron C., Hinckel P., Jouvet M. (1997). Afferent projections to the rat nuclei raphe magnus, raphe pallidus and reticularis gigantocellularis pars alpha demonstrated by iontophoretic application of choleratoxin (subunit b). J. Chem. Neuroanat..

[bib26] Hökfelt T., Arvidsson U., Cullheim S., Millhorn D., Nicholas A.P., Pieribone V., Seroogy K., Ulfhake B. (2000). Multiple messengers in descending serotonin neurons: localization and functional implications. J. Chem. Neuroanat..

[bib27] Huma Z., Du Beau A., Brown C., Maxwell D.J. (2014). Origin and neurochemical properties of bulbospinal neurons projecting to the rat lumbar spinal cord via the medial longitudinal fasciculus and caudal ventrolateral medulla. Front. Neural Circuits.

[bib28] Hung K.C., Wu H.E., Mizoguchi H., Leitermann R., Tseng L.F. (2003). Intrathecal treatment with 6-hydoxydopamine or 5,7-dihydroxytryptamine blocks the antinociception induced by endomorphin-1 and endomorphin-2 given intracerebroventricularly in the mouse. J. Pharmacol. Sci..

[bib29] Jankowska E., Hammar I., Chojnicka B., Hedèn C.H. (2000). Effects of monoamines on interneurons in four spinal reflex pathways from group I and/or group II muscle afferents. Eur. J. Neurosci..

[bib30] Jones S.L., Light A.R. (1990). Termination patterns of serotoninergic medullary raphespinal fibers in the rat lumbar spinal cord: an anterograde immunohistochemical study. J. Comp. Neurol..

[bib31] Jones S.L., Light A.R. (1992). Serotoninergic medullary raphespinal projection to the lumbar spinal cord in the rat: a retrograde immunohistochemical study. J. Comp. Neurol..

[bib32] Kachidian P., Poulat P., Marlier L., Privat A. (1991). Immunohistochemical evidence for the coexistence of substance P, thyrotropin-releasing hormone, GABA, methionine-enkephalin, and leucin-enkephalin in the serotonergic neurons of the caudal raphe nuclei: a dual labeling in the rat. J. Neurosci. Res..

[bib33] Kilo S., Schmelz M., Koltzenburg M., Handwerker H.O. (1994). Different patterns of hyperalgesia induced by experimental inflammation in human skin. Brain.

[bib34] Lee H.G., Kim W.M., Kim J.M., Bae H.B., Choi J.I. (2015). Intrathecal nefopam-induced antinociception through activation of descending serotonergic projections involving spinal 5-HT_7_ but not 5-HT_3_ receptors. Neurosci. Lett..

[bib35] Liebeskind J.C., Guilbaud G., Besson J.M., Oliveras J.L. (1973). Analgesia from electrical stimulation of the periaqueductal gray matter in the cat: behavioral observations and inhibitory effects on spinal cord interneurons. Brain Res..

[bib36] Marlier L., Rajaofetra N., Poulat P., Privat A. (1990). Modification of serotoninergic innervation of the rat spinal cord dorsal horn after neonatal capsaicin treatment. J. Neurosci. Res..

[bib37] Millan M.J. (2002). Descending control of pain. Prog. Neurobiol..

[bib38] Nitzan-Luques A., Devor M., Tal M. (2011). Genotype-selective phenotypic switch in primary afferent neurons contributes to neuropathic pain. Pain.

[bib39] Nitzan-Luques A., Minert A., Devor M., Tal M. (2013). Dynamic genotype-selective “phenotypic switching” of CGRP expression contributes to differential neuropathic pain phenotype. Exp. Neurol..

[bib40] Normandin J.J., Murphy A.Z. (2008). Nucleus paragigantocellularis afferents in male and female rats: organization, gonadal steroid receptor expression, and activation during sexual behavior. J. Comp. Neurol..

[bib41] Oliveras J.L., Besson J.M., Guilbaud G., Liebeskind J.C. (1974). Behavioral and electrophysiological evidence of pain inhibition from midbrain stimulation in the cat. Exp. Brain Res..

[bib42] Paxinos G., Watson C. (2005). The Rat Brain in Stereotaxic Coordinates.

[bib43] Potrebic S.B., Fields H.L., Mason P. (1994). Serotonin immunoreactivity is contained in one physiological cell class in the rat rostral ventromedial medulla. J. Neurosci..

[bib44] Rahman W., Suzuki R., Webber M., Hunt S.P., Dickenson A.H. (2006). Depletion of endogenous spinal 5-HT attenuates the behavioural hypersensitivity to mechanical and cooling stimuli induced by spinal nerve ligation. Pain.

[bib45] Rivot J.P., Chaouch A., Besson J.M. (1980). Nucleus raphe magnus modulation of response of rat dorsal horn neurons to unmyelinated fiber inputs: partial involvement of serotonergic pathways. J. Neurophysiol..

[bib46] Rivot J.P., Chiang C.Y., Besson J.M. (1982). Increase of serotonin metabolism within the dorsal horn of the spinal cord during nucleus raphe magnus stimulation, as revealed by in vivo electrochemical detection. Brain Res..

[bib47] Skagerberg G., Bjorklund A. (1985). Topographic principles in the spinal projections of serotonergic and non-serotonergic brainstem neurons in the rat. Neuroscience.

[bib48] Steinbusch H.V.W. (1981). Distribution of serotonin-immunoreactivity in the central nervous system of the rat - cell bodies and terminals. Neuroscience.

[bib49] Suzuki R., Rygh L.J., Dickenson A.H. (2004). Bad news from the brain: descending 5-HT pathways that control spinal pain processing. Trends Pharmacol. Sci..

[bib50] Todd A.J. (2010). Neuronal circuitry for pain processing in the dorsal horn. Nat. Rev. Neurosci..

[bib51] VanderHorst V.G.J.M., Ulfhake B. (2006). The organization of the brainstem and spinal cord of the mouse: relationships between monoaminergic, cholinergic, and spinal projection systems. J. Chem. Neuroanat..

[bib52] Vertes R.P., Kocsis B. (1994). Projections of the dorsal raphe nucleus to the brainstem:PHA-L analysis in the rat. J. Comp. Neurol..

[bib53] Wang Q.P., Nakai Y. (1994). The dorsal raphe: an important nucleus in pain modulation. Brain Res. Bull..

[bib54] Wei F., Dubner R., Zou S., Ren K., Bai G., Wei D., Guo W. (2010). Molecular depletion of descending serotonin unmasks its novel facilitatory role in the development of persistent pain. J. Neurosci..

[bib55] West S.J., Bannister K., Dickenson A.H., Bennett D.L. (2015). Circuitry and plasticity of the dorsal horn – toward a better understanding of neuropathic pain. Neuroscience.

[bib56] Woolf C.J., Salter M.W. (2000). Neuronal plasticity: increasing the gain in pain. Science.

[bib57] Yamazaki N., Umeno H., Kuraishi Y. (1999). Involvement of brain serotonergic terminals in the antinociceptive action of peripherally applied calcitonin. Jpn. J. Pharmacol..

[bib58] Yang J., Bae H.B., Ki H.G., Oh J.M., Kim W.M., Lee H.G., Yoon M.H., Choi J.I. (2014). Different role of spinal 5-HT(hydroxytryptamine)_7_ receptors and descending serotonergic modulation in inflammatory pain induced in formalin and carrageenan rat models. Br. J. Anaesth..

[bib59] Ziegler E.A., Magerl W., Meyer R.A., Treede R.D. (1999). Secondary hyperalgesia to punctate mechanical stimuli. Central sensitization to A-fibre nociceptor input. Brain.

